# Qualitative evaluation of the Teenage Mothers Project in Uganda: a community-based empowerment intervention for unmarried teenage mothers

**DOI:** 10.1186/1471-2458-13-816

**Published:** 2013-09-08

**Authors:** Joanne N Leerlooijer, Arjan ER Bos, Robert AC Ruiter, Miranda AJ van Reeuwijk, Liesbeth E Rijsdijk, Nathan Nshakira, Gerjo Kok

**Affiliations:** 1 Division of Human Nutrition, Wageningen University, Wageningen, The Netherlands; 2 Department of Work and Social Psychology, Maastricht University, Maastricht, The Netherlands; 3 Rutgers WPF, Utrecht, The Netherlands; 4 School of Psychology, Open University, Heerlen, The Netherlands; 5 Windesheim University of Applied Sciences, Windesheim Honours College, Zwolle, The Netherlands; 6 Uganda Christian University, Mukono, Kampala, Uganda

**Keywords:** Empowerment, Stigma, Teenage pregnancy, Qualitative evaluation, Agency, Community, Social change

## Abstract

**Background:**

A large proportion of unmarried teenage mothers in Uganda face physical, psychological, and social problems after pregnancy and childbirth, such as obstetric complications, lack of education, and stigmatisation in their communities. The Teenage Mothers Project (TMP) in Eastern Uganda empowers unmarried teenage mothers to cope with the consequences of early pregnancy and motherhood. Since 2000, 1036 unmarried teenage mothers, their parents, and community leaders participated in economic and social empowerment interventions. The present study explored the changes resulting from the TMP as well as factors that either enabled or inhibited these changes.

**Methods:**

Semi-structured interviews (*N* = 23) were conducted with former teenage mothers , community leaders, and project implementers, and lifeline histories were obtained from former teenage mothers (*N* = 9). Quantitative monitoring data regarding demographic and social characteristics of teenage mother participants (*N* = 1036) were analysed.

**Results:**

The findings suggest that, overall, the TMP seems to have contributed to the well-being of unmarried teenage mothers and to a supportive social environment. It appears that the project contributed to supportive community norms towards teenage mothers’ position and future opportunities, increased agency, improved coping with early motherhood and stigma, continued education, and increased income generation by teenage mothers. The study findings also suggest limited change in disapproving community norms regarding out-of-wedlock sex and pregnancy, late active enrolment of teenage mothers in the project (i.e., ten months after delivery of the child), and differences in the extent to which parents provided support.

**Conclusions:**

It is concluded that strengths of the community-based TMP seem to be its socio-ecological approach, the participatory planning with community leaders and other stakeholders, counselling of parents and unmarried teenage mothers, and the emphasis on education and income generation. The project can improve by earlier active participation of unmarried pregnant adolescents and increased support for parents.

## Background

Even though fertility rates among adolescents aged 15–19 in sub-Saharan Africa have declined over the past decades, a large proportion of women delivers a child during adolescence [1]. Age of marriage has been increasing in most countries, whereas adolescents have remained sexually active and few of them consistently use contraceptives. This has resulted in an increased prevalence of out-of-wedlock pregnancies in adolescents [2]. Uganda is one of the countries in sub-Saharan Africa with high rates of teenage pregnancy: one out of four females aged 15–19 gives birth to a child and the proportion of unplanned pregnancies has increased between 2001 and 2011 [3]. Out-of-wedlock teenage pregnancy has major physical, social, and psychological consequences. In addition to the need to cope with motherhood, many unmarried teenage mothers face stigma, lack of schooling, and livelihood insecurity [4-9]. The psychological and social consequences of out-of-wedlock motherhood can be addressed in interventions targeting teenage mothers combined with interventions focusing on their social environment. Several studies and reviews suggest a comprehensive, community-based, and socio-ecological approach [4,9-13].

A comprehensive, socio-ecological approach that focuses on relevant intrapersonal, interpersonal, community, organisational, national, and global levels of influence and interaction, moves beyond change of individual behaviours. It recognises and addresses the structural contexts which shape and limit people’s agency and therefore ability to act [14,15]. In an empowerment approach, these socio-ecological levels of influence are incorporated, whereby empowerment is a result of the interaction between individual *agency* (i.e., the freedom to do whatever one needs to do to achieve goals or values that one views as important) and *opportunity structure* (i.e., the broader institutional, social, and political context of formal and informal rules and norms within which actors pursue their interests) [16,17].

Increasingly, evidence about socio-ecological interventions is generated by HIV prevention and care strategies [18,19], HIV-stigma reduction interventions [20,21], intimate-partner violence interventions [22,23], and gender and microfinance interventions [24]. However, there is a need for empirical studies, to measure the effect of socio-ecological interventions in general [18,24] and of reproductive health interventions targeting unmarried teenage mothers in particular [13,25]. Evaluation of complex, socio-ecological interventions, presents a number of challenges, including restricted opportunities for randomisation, inadequate effect attribution to specific interventions, and limited control over implementation. Qualitative evaluation research can contribute to the identification of changes that result from specific intervention elements, which could be further explored in quantitative impact evaluation [26].

To contribute to the evidence-base of socio-ecological reproductive health interventions, we conducted a qualitative evaluation exploring the effects of a community-based intervention in Eastern Uganda. The intervention under study is the Teenage Mothers Project (TMP) that aims to create a supportive social environment and to address consequences of out-of-wedlock pregnancy and motherhood among unmarried teenage mothers.

### The intervention: the Teenage Mothers Project

The TMP is implemented in the Manafwa district which is populated by the Bugisu tribe and situated on the Kenyan border. The district, approximately 360,000 inhabitants, comprises 164 predominantly rural parishes. The TMP participants originate from 52 parishes in the district. Most inhabitants are farmers and some are traders. Young people are often unemployed or are engaged in temporary or seasonal work in the informal economy. Socioeconomic standards and literacy rates are generally low.

The participatory project planning was guided by a planning group consisting of staff and volunteers of the community based organisation African Rural Development Initiatives (ARDI), a health promotion professional, and governmental, religious, and tribal leaders and unmarried teenage mothers from the Manafwa district [27]. The planning group discussed suggestions of teenage mothers and community leaders to address certain needs, checked these with the health promotion professional, and implemented the selected interventions on a small scale. If monitoring data showed acceptance by implementers and indicated change among beneficiaries, the activities were implemented on a larger scale. The project started in 2000, based on the suggestion of unmarried teenage mothers to support them with a female goat that would supply them with milk to support their child, with offspring that could be traded for a cow (approximately 25 goats), and that would give them respect in their family and community.

The iterative project design was guided by the Intervention Mapping framework to ensure that the intervention was based on theory and evidence and to maximise the likelihood of effectiveness [28]. Intervention Mapping is a stepwise planning framework and describes the iterative process of behaviour change programme development from problem identification to problem solving or mitigation. The first step was to conduct a needs assessment, revealing that out-of-wedlock teenage pregnancy, stigma, and violation of the rights of unmarried mothers, and unawareness of its magnitude and consequences, were a major problem in the communities in the Manafwa district [29]. It was decided to address the consequences of out-of-wedlock teenage pregnancies by intervening among various actors: the unmarried teenage mothers and their parents or guardians (referred to as parents in the remaining of this paper), school administrators, religious leaders, tribal leaders, governmental leaders on village, sub-county, district and national level, journalists, and the community at large. Even though the needs assessment showed that many teenage mothers had to cope with the father of their child not taking responsibility, teenage mothers were hesitant to disclose the father’s name because they feared harassment or, in case of a loving relationship, feared that he would be accused of defilement. It was therefore decided not to include the children’s fathers as target group in the TMP.

The overall programme outcome was to improve the psychological and social well-being of unmarried teenage mothers in the Manafwa district by increasing their decision making power (*agency*) and creating a supportive environment (*opportunity structure*). Behavioural outcomes for teenage mothers included effective coping with stigma and with motherhood, continuation with education, income generation, having no or protected sex, and advocacy for their rights. Coping was addressed through emotion-focused strategies (e.g., regulating negative emotions resulting from stigma or motherhood) and problem-focused strategies (e.g., reconciliation with parents) [30,31]. Environmental outcomes included increased support for continued education and increased care for the unmarried teenage mother and her child by various environmental actors including parents and community leaders.

To address the behavioural and environmental outcomes, five intervention components were developed: community sensitisation, teenage mothers support groups, livelihood (continued education and income generation), counselling, and advocacy. Behavioural and environmental change methods for these five components included participatory planning [32], modelling [33], mobilisation of social networks [34], persuasive communication [35], entertainment education [36], advocacy [37], guided practice [38], provision of information about relevant others’ approval [39], and social action [37]. See Bartholomew et al. [28] for an overview of behavioural and environmental change methods. The intervention was adopted and implemented by trained community based volunteers, ARDI staff and community leaders. Table 1 provides an overview of the intervention components, beneficiaries, and deliverers of the TMP. The planning and a more elaborate description of the intervention are described by Leerlooijer et al. [27].

**Table 1:** Components, beneficiaries, and deliverers of the Teenage Mothers Project

**Intervention components**	**Description**	**Beneficiaries**		**Deliverers**
		**Unmarried teenage mothers**	**Environmental actors**	
1. Community awareness raising	Awareness raising during community meetings, including goats giving ceremonies in the parish of approximately 30 teenage mothers, attended by community leaders and decision makers.	*n.a.*	Community members	Community based volunteers
			Parents	
			National and district leaders	ARDI staff
			Clan, religious, village, sub-county leaders	Clan, religious, village, sub-county leaders
	Intervention activities: speeches by influential (national) leaders, discussion about out-of-wedlock teenage pregnancy, testimonies of teenage mothers and their parents, and songs and theatre plays (edutainment).			
			School administrators	
2. Teenage mothers support group	A group consists of teenage mothers from a particular parish in the project area. The groups focus on social support, advocacy, income generation and sexual and reproductive health and rights (SRHR) education.	Unmarried teenage mothers	*n.a.*	Community based volunteers
				Clan, religious, village, sub-county leaders
3. Livelihood	Includes continued (formal) education of the teenage mothers and income generation in the support groups and individually (i.e., goat rearing).	Unmarried teenage mothers	School administrators	Community based volunteers
				Clan, religious, village, sub-county leaders
4. Counselling	Aimed at coping with stigma, reconciliation of relationships between parents and teenage mothers, and advice for continued education.	Unmarried teenage mothers	Parents	ARDI staff (counsellors)
				Community based volunteers
5. Advocacy	Aims to change and/or implement policies and legislation and to create more awareness and discussion about the well-being of unmarried teenage mothers in Uganda.	*n.a.*	National, district, and community leaders	ARDI staff
				Unmarried teenage mothers
				Journalists

Between March 2000 and March 2012, 1036 unmarried teenage mothers from 52 parishes in the Manafwa district had been participating in the TMP. Almost all teenage mothers in the project area had enrolled in the project, except for a small number of adolescents in relatively wealthy families who did not meet the enrolment criteria. The project is still being implemented at the time of writing (August 2013).

### The present study

This paper describes a retrospective, qualitative effect evaluation of the TMP in Eastern Uganda, aiming to explore the effects of the project on the social environment and on the psychological and social well-being of unmarried teenage mothers in rural communities in Eastern Uganda. Twenty-three semi-structured interviews were conducted with former teenage mothers, community leaders, staff of the implementing organisation ARDI, and community based volunteers.

## Methods

### Respondents and sampling procedure

The study was conducted in March 2012 in the Manafwa district by a research team consisting of the principal researcher and two research assistants. The data collection methods included semi-structured interviews, a lifeline history methodology, and monitoring sheets. Interviews were conducted with 23 respondents. Nine interviews were conducted with former teenage mothers who were selected from the teenage mothers who had ever participated in the TMP. The mean age of the nine respondents was 24.1 years old (*SD* = 4.5; range = 18–32). The respondents were selected aiming at a mixture of respondents who varied in age, marital status, location, and the extent to which they had continued their education and had generated income. We continued until data saturation was reached. In addition to former teenage mothers, various relevant actors in the social environment of teenage mothers participated in the interviews: one male parent, one male community elder, two male governmental village leaders, two male religious leaders, two school administrators (one female and one male), four staff of the community based organisation ARDI (one female, three male), and two community based volunteers (one male and one female). The respondents were selected based on the following criteria: related to at least one of the former teenage mothers (e.g., school administrator of a school that was attended by one of the respondents) and comfortable expressing themselves in English. Prior to data collection, the number of interviews with actors in the social environment was decided upon and was limited by availability of time and resources. All interview respondents were residents of eight parishes in the project area.

### Procedure

The study was approved by the Ethical Committee Psychology (ECP) of Maastricht University in the Netherlands and the study adheres to the RATS guidelines for qualitative research. The chief administrative officer of the Manafwa district in Uganda provided permission to conduct the interviews. Monitoring sheets with demographic and social characteristics of the teenage mothers had been completed since March 2000 and were entered in data processing software. In March 2012, the most recent information regarding marital status, number of children, continuation with education, highest level of education, and data on income generation was collected by the ARDI project officer from the community based volunteers.

Interviews were conducted by the principal investigator, a Dutch female aged 34 (first author), and by two research assistants: a Dutch male aged 43 and a female aged 24 from the Bugisu tribe living in the Manafwa district. The research assistants were selected based on their interview skills and language. The interviews with former teenage mothers, two parents, and one community based volunteer were conducted by the Mugisu female. The interviews with community leaders were conducted by the Dutch male. The interviews with organisation staff were conducted by the Dutch principal investigator. The Dutch principal investigator and Dutch male research assistant conducted interviews with respondents who were comfortable to respond in English. The Mugisu interviewer was able to accommodate respondents who were not familiar with English and who felt more comfortable expressing themselves in the vernacular language Lugisu. A two day training of the research assistants was conducted aiming at explanation and planning of the research, at testing, practicing and adaptation of the methodology, and (back-) translation of the semi-structured interview guides.

Once recruited, interview respondents were informed about the study’s purpose and procedure. Interviews were tape-recorded with consent of the respondents. Verbal informed consent to participate in the interview was obtained and tape-recorded. All respondents were informed that they could withdraw from the study at any time. The interviews with most respondents were held at their homes, whereas the interviews with the school administrators were held at their respective schools. The interviewer asked to use a quiet room or space to conduct the interviews.

Semi-structured interview guides were designed and were based on empowerment theories, exploring individual and environmental changes resulting from the TMP and its supporting and inhibiting factors. The interviews included similar topics for all respondents. The topics were based on three degrees of empowerment [40], i.e., *existence of choice* (e.g., ‘How would you describe the TMP?’), *use of choice* (e.g., ‘Who influenced you (or a teenage mother) in the decisions that you (or a teenage mother) are taking?’), and *effectiveness of choice* (e.g., ‘What has been the most significant change of the project for you /unmarried teenage mothers?’ and ‘What can be improved in the project?’). Questions in the interview guides for teenage mothers were directed at their personal experiences as well as their perceived experiences of other teenage mothers. The interview guides for the other respondents were directed at their personal experiences and their perceived changes among community members and among teenage mothers.

**Figure 1:**
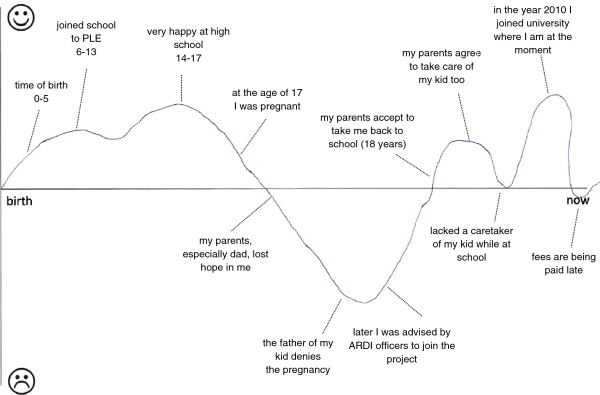
Example lifeline history of a former teenage mother.

The interviews with the former teenage mothers started with drawing of a lifeline history to facilitate interviewer-respondent dialogue about life events [41]. The interviewer introduced a sheet of paper with a single line printed across the landscaped page. At the left end of the line was the word “birth”; and the right end was the word “now” (see Figure 1). Instructions regarding the lifeline were read aloud to the respondent.

### Data analysis

The interviews were transcribed verbatim and the paper sheets with lifeline histories were transformed into digital sheets. The transcript interview data and the digital sheets were processed with ATLAS.ti qualitative software. The data were analysed using a general inductive approach [42]. After data cleaning and preparation, the principal investigator carefully read the text and analysed the lifeline histories. The principal investigator used coding to identify categories and built a model based on the research questions and the empowerment approach (i.e., agency and opportunity structure). The categories included intrapersonal and external factors that either contributed to or inhibited psychological and social well-being of unmarried teenage mothers. Subsequently, sub-categories were identified. For example, the category ‘coping’ was divided into the sub-categories ‘coping with pregnancy and motherhood’, ‘coping with stigma’, and ‘marriage as coping strategy’. Optional quotes were highlighted in the data and the (sub-)categories were continuously revised and refined. After finalising the categorisation, preliminary findings were documented and discussed during a consensus meeting of ARDI staff members and a former teenage mother. Descriptive and frequency analyses were conducted using the quantitative software package SPSS 19.0 to describe the demographic and social characteristics of the teenage mother participants.

## Results

### Demographic and social characteristics of TMP participants

Between March 2000 and March 2012, 1036 unmarried teenage mothers had been participating in the project. Demographic and social characteristics of the participants are provided in Table 2. The majority of teenage mothers had a Catholic background, a minority Muslim, and the remaining Protestant. In March 2012, the mean age of the 1036 participants was 20.5 years (range 14–33) and a majority of 57.8% (age > 16) was married at that time. The quantitative data indicated that almost half of the 1036 teenage mothers was still participating in the project at the time of the research (March 2012). The other participants either retracted from the project because of continued education, employment, business, or marriage, or because they passed away. The mean age of participants was 15.75 years (*SD* = 1.22; range 10–18) when they gave birth to their child and 10 months older (16.53 years; *SD* = 1.36) when they joined the project.

**Table 2:** Demographic and social characteristics of teenage mother participants

**Characteristic**	***N*** **= 1036**	**%**
**Religious background (*N*** **= 1023)**		
Catholic	749	73.2
Protestant	238	23.3
Muslim	36	3.5
**Marital status (*N*** **= 1022)**		
Married	591	57.8
Unmarried	431	42.2
**Participation in TMP**		
Passed away	17	1.6
Retracted from project because of continued education, employment, business, or marriage	512	49.4
Participating in project	507	49.0
Highest level of education* (*N* **= 960)**		
Primary education (level 1–7)	385	40.1
At least lower secondary education (level 1–4)	468	48.8
At least higher secondary education (level 5–6)	88	9.2
Higher education or university	19	2.0

* These figures include both project participants who attended education and participants who resigned from education.

### Qualitative findings

The overall aim of the TMP was to address psychological and social consequences of out-of-wedlock pregnancy and motherhood among unmarried teenage mothers in the Manafwa district by increasing unmarried teenage mothers’ decision making capacity and autonomy (*agency*) and by creating a supportive environment (*opportunity structure*). An inductive analysis of the qualitative interview findings resulted in five result areas: social norms, agency, coping, education, and income generation of the unmarried teenage mothers. In general, the ARDI staff, community based volunteers and community leaders reported in a more common and more positive way about the project’s results than the former teenage mothers did. The ARDI staff members estimated that approximately 60-80% of the 1036 participants in the TMP had been able to benefit from the project through continued education, increased economic benefits, improved relationships, and/or effective coping. Even though it appears that quality of life of a large majority of participants had improved after joining the project, the interviews data suggest that they also still had to cope with challenges such as lack of resources for school fees to continue education or lack of employment after finishing a certain level of education.

#### ***Social norms***

Based on the interview data it seems that community norms with regard to the unmarried teenage mothers and the project had changed between 2000 and 2012, whereas norms with regard to out-of-wedlock sex and pregnancy had remained unchanged. It appears that before the project started, parents and community members and leaders generally had a negative attitude towards unmarried teenage mothers, influenced by deeply embedded cultural and religious moral beliefs regarding out-of-wedlock pregnancy and sex. The interviews seem to indicate that these beliefs did not change in the course of the project. For example, a pastor of a Christian church refers to out-of-wedlock sex and pregnancies as mistakes:

When somebody [*unmarried teenage mother*] has gone back to school and realises “This is what caught me to be helped and go back”, it changes that person completely and they learn from the former mistakes. Because at times we learn by mistakes, we learn by the Spirit, we learn by the word of God. (Male pastor of a Christian church)

However, the response of the pastor also illustrates that despite the disapproval of teenage mothers’ out-of-wedlock sexual behaviour, the norms with regard to the position of and future possibilities for unmarried teenage mothers seem to have changed. The interview data suggest that these norms have become less stigmatising and more supportive for the teenage mothers. Instead of encouraging marriage and discontinuation of the girl’s education after out-of-wedlock pregnancy, it seems that parents had become increasingly supportive of delayed marriage and continued education. One of the former teenage mothers explained:

I used to feel that I am already second hand, why should I go back to school? Even my mother told me to get married. You know what happens in a polygamous family, when you get pregnant you get married. […] That project really helped me, even my mum got encouragement from it. Coz’ those people told her to take me back to school. Without, she had left me to go and get married because I was pregnant. (Former teenage mother, 23 years old, married)

However, the interview data also give the impression that not all community members, leaders, peer youth and parents had changed their opinion and behaviour. A school head master reported:

Then there are peer groups. Some have these insulting words: “You are married, what are you doing in school?” Because of that fear, some of them are abandoned. […] So they are pointing: “Look at that girl, she delivered, now what is she still doing?” […] It is believed that when she produces, her life has ended. So that societal attitude may again discourage those ones to continue to be in class. (Head master secondary school)

A first step towards change in social norms appeared to be the involvement and consultation of influential community leaders in finding solutions for problems in the community, out-of-wedlock pregnancies being one of them. This included awareness raising about the magnitude of the problem and convincing community leaders of the need to jointly support unmarried teenage mothers. Initially, the community leaders were resistant and it required considerable efforts to convince community leaders of the need of the project. The interviews indicated that the change of norms had been a lengthy process:

This particular project, the goats project, has opened people’s eyes. To really identify a problem that looked like it was hidden. People would not really come into realisation of this problem. […] And now, whatever the case, people are slowly, slowly coming to realisation, they are thinking ‘Eh, I think we have to do something about that’. (Male ARDI social worker)

The initiative of three governmental leaders to support teenage mothers support groups with a plot of land or a venue for their activities can be interpreted as an indication that attitudes of at least some of the governmental leaders had changed. Once influential community leaders were supportive to the project and its approach, they were increasingly involved in community sensitisation. The goats giving ceremonies appeared to be a significant strategy to sensitise the community. It seems that testimonies of parents of ‘successful’ unmarried teenage mothers about their own coping process were among the effective strategies to influence other parents. An ARDI staff member reported about a goats giving ceremony:

There was one of the mothers of the children who succeeded. She came and talked here, before the rest of the people, and gave testimony of how she has supported this girl and how she is now very proud of her daughter. She was given time to really talk and I was really very impressed with her speech. And everybody was really listening. And of course those were now new parents and new girls and they were really listening. […] And now the girl is really a hope for the entire family. And I feel that those stories are really inspiring. (Male ARDI social worker)

It was reported that in later phases in the project, community norms also seem to have changed because of the successes of various teenage mothers in education and income generation.

#### ***Agency***

The interviews give the impression that decision making freedom of unmarried teenage mothers in the TMP has to certain extent improved. It seems that particularly as a result of changed social norms and increased social support, continued education, and income generation, teenage mothers gained more freedom to do whatever they needed to do to achieve their goals (agency). It seems that improved self-confidence and improved autonomy had contributed to increased agency. It appears that self-confidence of teenage mothers generally had been very low during pregnancy and early motherhood, but that this had improved as a result of counselling, participation in the teenage mothers’ support groups and a more supportive environment. The section about coping further describes how belonging to a group contributed to their level of coping and self-confidence. Confidence of teenage mothers was also reflected in their capacity and responsibility to be an example for other teenage mothers and their parents. A former teenage mother shared her vision:

You can see where I have reached: I am now in campus. And I can also advise others. I advise parents who have their girl who produce at an early age. I advise them to take them back to school. (Former teenage mother, 22 years old, unmarried)

On first sight it seems that teenage mothers’ autonomy had also increased, particularly as a result of continued education and income generation, providing them with financial resources and with knowledge and skills to take care of themselves. However, further analysis of the data suggests that teenage mothers also remained to be dependent on the decisions of their father or their husband. After asking whether she could take her own decisions, a former teenage mother reported:

I can. Though I still depend on my parents. But I can. […] Right now I take decisions myself, because I know what is good and what is bad. Generally I know what to do. (Former teenage mother, 22 years old, unmarried)

It seems that if teenage mothers’ goals correspond with the goals of their parents or husbands, there is no conflict. However, non-correspondence of goals can challenge teenage mothers to do what they need to do to achieve their goals. It appears that limited agency of teenage mothers strongly relates to the extent their social environment and other external factors were supportive. This is illustrated by an ARDI staff member:

In my view, there are limitations in these girls making decisions. Not because they can’t make decisions, but sometimes they are influenced by circumstances around them. For example, you may want to be a person of principle, but there are certain things that make you drop your principles. For example, hunger. Hunger can sometimes force someone change their principles. (Male ARDI social worker)

The general impression that agency of teenage mothers had increased as a result of the project, was reflected in stories about improved care for their child, marriage with ‘better’ husbands, continuation with education and prevention of early marriage and of transactional sex. Increased agency of teenage mothers seems to strongly relate to the extent they were able to generate their own income to care for their child, to pay their own school fees, and to purchase daily necessities. Increased agency also seemed to be related to the extent teenage mothers could decide when and whom they would marry. It appears that female teenagers who were better educated were more attractive for males, giving the teenage mothers more opportunities to choose a husband. The interviews indicated that this also increased the chance to get married to a husband who would care well for them and to a more equal relationship. One of the former teenage mothers reported:

I gave birth in 1999, but through counselling and sensitisation, I managed to stay without giving birth for a long time, until I gave birth to Rose in 2004. And out of that I have managed to look for a husband who can take good care of me. […] And out of that, I have been able to go back to school. And now I earn my own money. (Former teenage mother, 32 years old, married)

According to respondents (community leaders, community based volunteers and ARDI staff), the number of early marriages and first and secondary pregnancies among teenage girls had decreased. The interviewed former teenage mothers attributed these declining figures to a decrease in (transactional) sex, which in turn would be a result of increased economic autonomy. One of them reported how increased income generation can prevent transactional sex:

When you are employed somewhere, even if you get little amount of money, it can help you. You can be getting 200,000 shillings *[i.e., US$ 78]*, you are able to buy clothes and take care of your child. But if you don’t have anything, you cannot be okay, because you will be overthinking about how to make life go on. Because it is through those challenges that someone can get problems in the process of making ends meet, like having sex to get some money. Yet, he will end up giving you only 1,000 – 2,000 shilling *[i.e., US$ 0.38 – 0.78]*. So it is better I get something to do for myself than depending on someone. (Former teenage mother, 27 years old, boyfriend)

Finally, it appears that the element of the TMP whereby individual, marginalised, teenage mothers were brought together into a visible group of connected people, had been a crucial factor. The teenage mothers had become visible in a positive way through registration of a majority of the 52 support groups as community based organisations, in media stories and during community gatherings such as the goats giving ceremonies. Their visibility as a group of generally successful community citizens seems to have contributed to more supportive social norms and to the collective power and in turn collective and individual agency of the teenage mothers. The community elder reported about this:

These child mothers, like some of them had lost hope, they had no future. But those few recently, who changed their minds and went back to school, and have completed their schools, they are leaders. And I think that is the most significant change that we have seen around […]. They decided to go back to school, they have completed their school. I think that is the biggest achievement. Because somebody who was, who would be useless, is now a leader (Community elder).

#### ***Coping***

The interview data and lifeline histories give the impression that many unmarried teenage mothers had to cope with great emotional turmoil as they strived to cope with pregnancy, delivery and early motherhood, a changed future perspective, lack of support of the father of the child, stigma of community members and peers, and negative responses of their parents. The lifeline histories showed that the period between discovery of their pregnancy and their introduction to the TMP, had been among the most difficult periods in their lives. See for example the lifeline history in Figure 1. Between discovery of the pregnancy and enrolment in the TMP, the ARDI staff and community based volunteers provided emotional support and counselling to the a number of female adolescents and their parents. The monitoring data showed that the average time between delivery of the child and full participation in ARDI’s activities had been 10 months. This was partly a result of shame to become visible in their community as an unmarried teenage mother. One of the former teenage mothers reported in her lifeline history about the period just after her pregnancy:

I got that pregnancy in 1999. So that is when my life changed to be upside down. I wanted to abort but I had no advice about abortion. And my mother noticed it and told me ‘Don’t do anything’. But my life changed completely knowing that now I will not join school, I am now no longer still a girl. I am now a mother until I came to produce that girl. Now, the baby was sick and I had no help. Because the man, the pregnancy was just like accidentally. The man was not willing to help me. The man sometimes says that that baby is not his’ and he keeps on denying. (Former teenage mother, 29 years old, married)

The interviews give the impression that the TMP has contributed among a majority of teenage mothers to effectively cope with the abovementioned challenges. The ‘advice of ARDI’ (counselling by counsellors and volunteers) and the teenage mothers groups were mentioned as important contributions to their coping capacity. The interviews suggest that teenage mothers felt taken seriously, listened to and treated as a valuable person by the ARDI staff and volunteers. It appears that teenage mothers shared their feelings and experienced that they mattered to others, which seems to have increased their self-confidence. Before they joined the TMP, most teenage mothers did not belong to the group of adult women, nor to their fellow non-pregnant peers, whereas participation in the project resulted in belonging to a group of unmarried teenage mothers. One of the former teenage mothers reported:

Having this education, like counselling meetings and income generating activity meetings, have helped me change my life and I have learned a lot. (Former teenage mother, 32 years old, married)

It appears that the attitude and behaviour of parents had been an important determinant of teenage mothers’ emotional turmoil as well as in their effective coping. Parents either contributed to or inhibited teenage mothers’ well-being by either expelling them from home or allowing them back, by allowing them to delay their marriage, by giving them emotional support and forgiveness, and by providing them support in motherhood, income generation, and continued education. It appears that counselling of the parents as well as role model examples of other parents had been interventions that contributed to changed attitudes and behaviours of parents. One of the former teenage mothers reported:

I felt bad and I stayed home for a whole year. And even after delivery I did not feel the joy of having a child. It was not until the ARDI people started counselling me that I started feeling that life is a little fine. The counsellor from ARDI talked to me personally and I felt okay. I was given a goat from ARDI and they also talked to my parents to take me back to school. And my parents accepted and took me back for the nursing assistant course and I joined. So I was happy. (Former teenage mother, 24 years old, not married)

The father of a teenage mother explained how the TMP had encouraged him to change his behaviour:

As a parent I got annoyed, of course, I got shocked and decided to leave her for a full year at home as a punishment. […] And through ARDI programme I also got some guiding and counselling. […] In case it is a mistake, we should not regard them as mistreats or as wastage. But they are people. In case they listen to your advice as a parent and go back to school, you give them some support. (Father of a teenage mother)

However, it appears that a small proportion of teenage mothers found it difficult to cope with their new situation and applied ineffective coping strategies such as early marriage and transactional sex. Even though it appears that the number of early marriages gradually declined since the start of the TMP in 2000, ARDI staff members estimated that around 30-40% of the 1036 participants had married at an early age, some with the father of the child. Teenage mothers either decided themselves to get married or their parents encouraged them to get married. For example, one of the former teenage mothers reported:

It was difficult to take care of the child and I needed a helper. So I decided to get married. […] That child doesn’t belong to this man, that doesn’t help so much. But I decided to get married on my own. (Former teenage mother, married, 29 years old)

Despite the emphasis of the TMP on economic autonomy and on parental support, it appears that transactional sex has remained a coping strategy of a minority of teenage mothers who were in financial need. The social worker explained that some teenage mothers who were in boarding schools were sometimes not sufficiently financially supported by their parents and some of them used transactional sex to sustain their living.

#### ***Education***

All respondents unanimously perceived teenage mothers’ continued education as the project’s most significant change, because of the large number of teenage mothers who returned to school, and its positive effects such as increased autonomy, self-confidence, and income generation. A school head mistress illustrated this change:

The Teenage Mothers Project has helped teenage mothers to go back to school. Which is very good, because prior to that you would find that once a child gets pregnant, that is the end of the education. So it has created that sensitisation in the community that even giving birth to a child is not the end of the education. […] I have seen girls that have dropped out of school, being able to come back to school. Which means that the environment, the mentality that they were having, has changed. (Head mistress secondary school)

The monitoring data indicated that up to March 2012, 65.4% of the 1036 participants in the TMP had returned either to primary or secondary school. The data showed that a large majority had completed at least primary education and that 94 teenage mothers (9.1%) had attended vocational skills training, such as tailoring or administrative courses. Table 2 provides an overview of teenage mothers’ highest level of education.

The interview data indicated that the extent to which teenage mothers had continued education, seemed to depend on teenage mothers’ motivation, support of their parents, social norms regarding education, and the school environment. It appears that a majority of teenage mothers was motivated to continue education, determined by their goal to take good care of the child, the observation of other teenage mothers who had succeeded in school, their level of self-confidence and self-efficacy, and their increased awareness of the value of education because of the experience being expelled from school. It appears that stigmatising behaviour of peer students in the schools was one of the factors hampering teenage mothers’ motivation to return to school (see the section ‘1. Social norms’). Various activities in the TMP seem to have contributed to their motivation, of which individual counselling (‘advice’) was most frequently mentioned. A community based volunteer reported:

The girls in my group that I have seen, have taken my advice and they have gone back to school. I feel I am very proud of them and I feel the work I have done is not a waste of time because they listen and yield to advice. The success of these girls has helped me reach out to other parents. My work is easy because they now believe that giving birth at home is not the end of every good thing, they can still achieve more if they are supported. (Female community based volunteer)

However, it appears that not all teenage mothers had been motivated to continue their education, as was illustrated by a school head mistress:

Okay, I have known of examples, like of recent, a girl was here in this school. She had reported back from school. The environment under which she was living is a privilege. The parents wanted her at school, but herself she has opted to go and marry; her own decision. Not that her parents were talking her out of home. (Head mistress secondary school)

Parental support seemed to be the most important factor determining teenage mothers’ continuation with education. Their support included financial support (school fees, school uniforms, school lunches, stationary) and childcare support when their daughter attended school. It appears that the level of support varied among parents, largely depending on their attitudes towards education of unmarried teenage mothers. Parents had to choose between marriage (and receiving dowry) and providing support for education, partly influenced by community norms. The respondents indicated that the attitudes and behaviours of parents had changed as a result of family counselling, follow up visits to their homes, changed community norms with regard to continued education, and good performance of teenage mothers in school. One of the former teenage mothers described how her parents responded when they found out she was pregnant, and how they had changed as a result of counselling and her own efforts:

Now, when they found out I was pregnant, my parents lost hope in me, especially my dad. He felt like sending me away from his home because he felt I was disobeying him and that I was not seeing the value of education. […] John is the one who came up to home and talked to my dad. […] They called me and asked me if I wanted to go to school. I said “Me, I want”. He said “It is ok, but now who will take care of the kid?” But still I said “Me, I will go”. And my mum said “I will take care of the kid”. When the results came back, I had passed and I was very happy. It also gave him the morale that I was serious there. (Former teenage mother, 22 years old, unmarried)

It appears that parents who did not support their daughter’s education either did not perceive this as a priority or lacked sufficient financial resources. The interviews provided numerous examples of teenage mothers who had not been able to continue education because their parents lacked resources to financially support them.

The interview data suggest that a supportive school environment has contributed to continued education of many teenage mothers. According to ARDI staff, awareness raising and persuasion of school administrators had changed their attitudes and allowed unmarried teenage mothers to return to school. A school head master reported about his changed attitude and behaviour:

So in the course, they asked at the school ‘Can you allow them to continue?’ So I said, ‘One, our problem is that these are the problem students, who are supposed to be in class. So now when they are mothers, it may be difficult’. But with the effort and their advice, we now allow them to become part of us, back to students’ life. But we caution them that once in school, they remember that we do not allow them to be a mother again. (Head master secondary school)

#### ***Income generation***

The TMP also seems to have contributed to increased income generation by the teenage mothers, through individual and collective income generating activities. Individually, teenage mothers had generated income through goat keeping, business or employment. One of the former teenage mothers explained how effective goats’ management had enabled her to exchange goats for a cow:

The goat grew and it produced. Although it was in bad health, I tried with my small money. I treated it, it grew, it produced and I got my cow now. I exchanged that cow to a female cow and I got a calf. Yah, it has helped because I can now sell that calf. I can pay for my girl. […] This cow can produce milk and I sell. If it produces much and I sell and it remains, my child takes some of it, also to support their health. (Former teenage mother, 29 years old, married)

It appears that parental support was conditional for the success of goats rearing as they were co-responsible for the goat, often located at their property. Parents and teenage mothers with goats’ management knowledge and skills appeared to be more successful in goat management and income generation. A community based volunteer illustrated this:

Some parents are generally good and when the girls get the goat they protect it. And even when the girl has gone to school they feed the goats. While other parents are bad. Immediately the girl gets that goat, they sell it and use it for personal reason. And some do not feed the goats when the girls are in school. So you find that those with support are more successful than those without support. (Female community based volunteer)

It was estimated that approximately 15% of all the provided goats had died or was sold because of (urgent) expenditures such as health care or medicines. However, according to ARDI staff members, these numbers had gradually declined in the course of the project as a result of more intensive monitoring, a Farm Africa goats management training to ARDI staff and volunteers, and goats management contracts between ARDI, each new project participant, her parents and the respective village leader.

Collective income was generated through a variety of activities by the teenage mothers support groups, including pharmacy management, bee, poultry, cattle and pig farming, sale of local snacks or coffee plant seedlings, and cultivation and sale of tomatoes, maize and onions. Out of the 52 support groups, 13 groups had been actively involved in income generation and generated approximately 200,000 shillings (US$ 76) per year. The groups had independently initiated these activities by a small financial investment by each of the group members. Once the activities were successful, ARDI reinforced this by providing in kind support such as seedlings. A community based volunteer reported:

Whenever we plant onions we get money, in fact good money, out of it. And we buy maize and currently we have stocked it in here in our store. Now after some time we sell, if we get a girl who has failed to raise enough for school. And we make our contribution and we equally help their young ones in terms of treatment when the mothers are away for school. (Female community based volunteer)

The interviews seem to suggest that the success of the income generating activities mainly depended on the leadership of the community based volunteers, on the commitment of the teenage mothers, and the support of the community members and leaders by providing groups with a piece of land or venue.

## Discussion

The study described in this paper explored the effects of the TMP in rural communities in Eastern Uganda. The qualitative findings provide guidance for other socio-ecological community-based interventions, and particularly for interventions aiming at improved psychological and social well-being of unmarried teenage mothers [13,25]. The project seems to have contributed to various positive changes in the project participants, whereas there are also elements that could be improved.

The first noticeable finding appears to be a change towards more supportive social norms among many parents, community leaders, and community members with regard to the value and future opportunities of unmarried teenage mothers and with regard to the TMP. It seems that beliefs and norms with regard to premarital sex and pregnancy have remained unchanged among all project stakeholders, whereas social norms concerning future opportunities of teenage mothers have become more supportive towards delay of marriage and continued education. Although inhibiting norms were also recorded, this was not the dominant view. One of the elements that appears to have contributed to changed social norms is an intensive, laborious process of participation and persuasion of influential community leaders. Other research confirms the importance to involve people who are affected by the intervention in the change process: community participation increases community ownership, community capacity, and programme maintenance [43] and that that changing social norms regarding sensitive topics such as sexuality and out-of-wedlock motherhood can be challenging and can take considerable time [44]. Other project activities that seem to have contributed to changed norms include community awareness raising meetings attended by relevant stakeholders, testimonies of successful parents and teenage mothers, supportive attitudes and behaviour of community leaders, and counselling of parents aimed at reconciliation with their daughter and at creating support for continuation with school instead of early marriage. There is opportunity for the project to improve, particularly with regard to social norms among parents and among peer youth in schools. Even though social stigma has not been entirely alleviated, it appears that changed social norms have contributed to decreased stigma of and to improved support for the unmarried teenage mothers.

A second noticeable finding was the unanimous perception of respondents that the TMP had particularly contributed to continued education of unmarried teenage mothers. Before the project started, a large majority of teenage mothers did not continue their education, whereas the study showed that 65% had returned to school. Corresponding with other studies, the present study shows that education tends to delay female’s marriage, to contribute to family planning, to improve their access to income, and to increase their economic aspirations and success [45,46]. In general, schooling is highly valued in Eastern Uganda and many see it as a gateway to a better life, to hopes for employment, social mobility, and being a modern person [47]. Continued education appears to depend on teenage mothers’ motivation to attend education, a supportive school environment, parental support, and resources to pay school fees. The results give the impression that the TMP contributed to changed attitudes and behaviour of parents and school staff, particularly through awareness raising, persuasion and/or counselling. The present study suggests that lack of parental support is one of the factors that impede teenage mothers’ education, which is also reported in other research [48]. Lack of resources for school tuition is among the most important and prevalent reasons for discontinued education. Even though other studies showed considerable reduction of dropout of school through cash transfer interventions [49] or provision of uniforms [50], disadvantages of these interventions were the lack of support of out of school children (such as unmarried teenage mothers) and large financial inputs. Aiming at sustainability, the TMP has provided training, counselling and a relatively small microcredit (goat) that appear to have supported critical thinking and own responsibility of parents and teenage mothers, within safe social spaces to enable them to think and act in new ways [43]. The project can improve by intensifying support for parents to generate resources for school fees, uniforms, lunches, and stationary.

A third finding was the contribution of the TMP to income generation of teenage mothers. Both continued education and income generation seem to have contributed to teenage mothers’ agency and coping strategies. Reviews regarding the impact of microfinance and other livelihood interventions for women provide ambiguous results, which relates to context-specific factors [51,52]. The present study suggests that income generation has contributed to increased economic autonomy, resulting in improved care of the child, prevention of early marriage and of transactional sex, marriage with ‘better’ husbands, and financial contribution to their own school fees. Activities in the TMP that have contributed to increased economic autonomy included the goat rearing, practical skills building such as tailoring and administrative skills, and the income generating activities of the teenage mothers groups. Even though efforts have been implemented to decrease the number of goats that die or are sold [53], the project can still improve by providing training to project participants and intensified goats’ monitoring by ARDI staff and community based volunteers. The project can also improve by encouraging more groups to initiate income generating activities.

Fourth, it appears that counselling and sexual and reproductive health and rights eduction, in addition to continued education and income generation, generally contributed to improved coping of the teenage mothers with early motherhood, the lack of support of the father of the child, stigma of community members and peers, a changed future perspective, and negative responses of their parents. It seems however that the point of time of active engagement of the teenage mothers in the project (on average 10 months after delivery of the child), is too late and may have caused trauma which could have been prevented when the project would actively intervene just after discovery of the pregnancy. Other research recommends to provide psychological and social support to the unmarried mothers immediately after discovery of the pregnancy to prevent traumatic experiences and internalisation of stigmatising norms and to increase the likelihood of better adjustment of unmarried teenage mothers and their children after birth [54].

Finally, the findings suggest a reinforcing interplay between the teenage mothers’ improved agency and the support provided by the parents and community leaders and members (opportunity structure). Other studies that have assessed the impact of structural, comprehensive interventions have also reported this interaction between individual and environment [24,55]. On the one hand, the unmarried teenage mothers seem to gain confidence, continue education and generate income because of the support of their parents and community. On the other hand, social norms in the community appear to have changed partly as a result of the visibility and successes of the teenage mothers. Teenage mothers as a group have become visible as a result of their collective action in the support groups, in media, and in community meetings. This has resulted in individual agency and has contributed to change in community norms and decreased stigma, which is also described in other studies exploring the influence of an individual versus a group of individuals [56]. In addition, the number of unmarried teenage mothers in Uganda increased in the past decades as a result of increased marital age, increased out-of-wedlock sexual activity of adolescents, and low contraceptive use [3]. Even though remaining a minority group, it appears that the collective action contributed to an increased sense of belonging and power among the teenage mothers. The findings support an ecological approach of interventions targeting all relevant actors including the teenage mothers, parents, community members, school administrators, and other community leaders [10].

### Strengths and limitations

Our study has both strengths and limitations. A strength was that the study was conducted among a variety of respondents representing relevant stakeholders in the community-based TMP, incorporating a variety of opinions and experiences. Another

strength is the combination of quantitative and qualitative data about the changes that appeared to have occurred as a result of the TMP. Triangulation of data across methods (quantitative data, life line histories, and semi-structured interviews) and respondents and checking of preliminary findings during a consensus meeting contributed to credibility of the findings.

A limitation of the study was the small sample size of each group of respondents. This mostly applies for the group of parents who particularly influence the teenage mothers’ well-being. Another limitation was the selection criterion to include community respondents who were comfortable to express themselves in English: they are more likely to be better educated and have a higher socioeconomic background than respondents who were not comfortable to express themselves in English. This may have influenced the results of the study.

### Recommendations

The study results provide recommendations for future research and practice. Qualitative evaluation research of a multi-level ecological intervention is a complex process as not only single, individual changes are analysed, but also the result of the interactions between various actors and interventions. Future studies should consider the inclusion of a sufficient number of respondents in each group to reach data saturation and obtain reliable, comprehensive data.

In addition, the study suggests to use a comprehensive, multi-level approach to mitigate the consequences of out-of-wedlock motherhood: to target both teenage mothers as well as various actors in their social environment; to use a variety of interventions ranging from counselling and education to income generating activities; and to address the needs and assets of the diverse group of unmarried teenage mothers. The findings on social norm change suggest that planners of reproductive health and rights interventions should incorporate an approach that balances between pushing the boundaries to be able to address adolescents’ needs and rights, and involving the community and adapting to a community context to avoid resistance and lack of implementation.

## Conclusions

Concluding, from the retrospective, qualitative evaluation it appears that the community-based TMP in Uganda has contributed to change in social norms with regard to opportunities for unmarried teenage mothers, to increased confidence and autonomy, to continued education and income generation by a large number of unmarried teenage mothers, and to improved coping with challenges. Counselling of parents and teenage mothers and advocacy among and involvement of community leaders and members seem to be activities in the TMP that have particularly contributed to these changes. The project can improve by earlier intervention among teenage mothers to prevent traumatic experiences, and by intensifying the support for parents to be able to financially support their daughters.

## Competing interests

JNL is volunteer and board member of Adopteer een Geit (Adopt a Goat) Foundation. Adopteer een Geit Foundation has provided financial and technical support to the community based organisation African Rural Development Initiatives (ARDI). This manuscript is not funded by Adopteer een Geit Foundation nor ARDI. The other authors declare that they have no competing interests.

## Authors’ contributions

JNL carried out the study and drafted the manuscript. MR was involved in study design. RACR and GK were involved in drafting the manuscript. AERB and LER made major corrections to the manuscript. NN revised the manuscript. All authors read and approved the final manuscript.

## Authors’ information

JNL is board member of Adopteer een Geit (Adopt a Goat) Foundation in the Netherlands, partner organisation of ARDI.

## Pre-publication history

The pre-publication history for this paper can be accessed here:

http://www.biomedcentral.com/1471-2458/13/816/prepub
